# Tissue Injury and Related Mediators of Pain Exacerbation

**DOI:** 10.2174/1570159X11311060003

**Published:** 2013-12

**Authors:** Fumimasa Amaya, Yuta Izumi, Megumi Matsuda, Mika Sasaki

**Affiliations:** Department of Anesthesiology, Kyoto Prefectural University of Medicine, Kajiicho 465, Kamigyo-Ku, Kyoto 602-8566, Japan

**Keywords:** Peripheral sensitization, postoperative pain, primary afferent neuron.

## Abstract

Tissue injury and inflammation result in release of various mediators that promote ongoing pain or pain hypersensitivity against mechanical, thermal and chemical stimuli. Pro-nociceptive mediators activate primary afferent neurons directly or indirectly to enhance nociceptive signal transmission to the central nervous system. Excitation of primary afferents by peripherally originating mediators, so-called “peripheral sensitization”, is a hallmark of tissue injury-related pain. Many kinds of pro-nociceptive mediators, including ATP, glutamate, kinins, cytokines and tropic factors, synthesized at the damaged tissue, contribute to the development of peripheral sensitization. In the present review we will discuss the molecular mechanisms of peripheral sensitization following tissue injury.

## INTRODUCTION

Why does tissue injury cause pain? This is an important but difficult question to answer. Although nerve injury as a consequence of tissue damage produces pain, tissue injury does not always cause nerve damage. Tissue inflammation is important when considering the mechanism of pain after tissue damage. We know much about the biology of the neuronal response after tissue inflammation. Most of our knowledge, however, comes from experiments in which tissue was artificially inflamed by the external application of pathogens. Currently, surgical procedures are one of the most common causes of tissue damage. In most cases, surgical procedures are performed under sterile conditions. Most inflammation after surgery is thus aseptic, making it naturally different from infection. However, not much is known about the differences in the reactions of the sensory nervous system to aseptic injury and infection.

Observations on postoperative pain using animal models [1] demonstrated that pain hypersensitivity following tissue injury is strongly associated with activation of primary afferent neurons. The phenomenon of activation of primary afferent neurons by peripherally derived mediators is known as “peripheral sensitization”. In the present review, we will introduce peripherally derived pro-nociceptive mediators and discuss how they activate primary afferent neurons at the molecular level.

### Mediators Released from Damaged Cells Provoke Pain

Using a co-culture system of primary afferent neurons with keratinocytes, Cook *et al*. demonstrated that nociceptors generate action potentials immediately after the destruction of nearby keratinocytes [2]. This observation indicates that soluble factors released from damaged cells can directly activate neighboring primary afferents, acting in a paracrine fashion. Potassium ions, hydrogen ions, adenosine tri-phosphate (ATP) and glutamate are candidates for the algogenic factors released from the cytosol (Table **1**).

## ADENOSINE TRI-PHOSPHATE (ATP)

ATP exists abundantly in the cytoplasm as an energy source. Extracellularly, ATP regulates the activity of various kinds of cells, including nociceptors, *via *purinergic receptors [3]. P2X (ionotropic) and P2Y (metabotropic) purinergic receptors are present in the primary afferent neurons [4]. Among them, homometric P2X3 and heterometric P2X2/X3 receptors transmit acute nociceptive sensory information. Bath application of ATP produces an inward current in cultured dorsal root ganglion (DRG) neurons under voltage-gated conditions [5]. Injection of the P2X receptor agonist, alpha/beta-methyl ATP, induces mechanical allodynia and thermal hyperalgesia at the site of injection [6]. The purinergic receptor antagonist, PPADS, inhibits plantar incision-induced pain hypersensitivity [7]. Metabotropic P2Y1 and P2Y2 receptors are also located in primary afferent neurons [4]. Both P2Y1 and P2Y2 receptors are involved in the enhancement of primary afferent neuron excitability associated with tissue injury [8, 9].

## GLUTAMATE

Ionotropic receptors (NMDA, AMPA and kainite receptors) for glutamate are present in primary afferent neurons [10], with the cytosol having a high concentration of glutamate. Glutamate depolarizes cultured DRG neurons [11]. Injection of agonists for NMDA, AMPA and kainite receptors into the glabrous skin produces transient mechanical and thermal hyperalgesia [12]. Hyperalgesia after acute inflammation resulting from formalin application is attenuated by glutamate receptor antagonists [13]. Apart from passive release from damaged tissue, glutamate is actively secreted from primary afferent neurons in response to electrical stimulation or capsaicin treatment [14].

## PROTONS AND POTASSIUM

The intracellular environment is slightly more acidic (pH=7.0-7.2) than the extracellular compartment [15]. Acid sensing ion channel (ASIC) 3 is a receptor that senses low pH in the peripheral nervous system. ASIC3 is activated by an acidic environment (pH=7.0) [16]. Genetic knockdown or pharmacological inhibition of ASIC3 reduces incision-induced pain hypersensitivity [17]. ASIC3 is believed to be responsive to chest pain resulting from ischemic cardiac tissue damage [16]. 

Potassium is one of the major intracellular cations. The difference in potassium concentration between the intra- and extra-cellular fluids determines the voltage potential of cellular membranes. There are several classes of voltage-gated potassium channels in the primary afferent neurons controlling neuronal excitability [18]. Injection of potassium into the dentinal cavity depolarizes the primary afferent fibers [19] and produces ongoing pain.

### Nociceptive Molecules as the Initial Step of Inflammation

Bleeding and coagulation due to tissue injury are closely associated with the initiation of inflammation [20]. Intra-dermal injection of platelets and leukocytes produces axon reflex erythema and acute pain responses in man. Kallikrein released during coagulation produces bradykinin, a strong algogenic factor. Degranulation of activated mast cells results in the release of proteases, cytokines, serotonin and histamine into the extracellular space. These substances sensitize primary afferent neurons to produce pain hypersensitivity [21] (Table **2**).

## BRADYKININ

Kinins, including the nonapeptide bradykinin and decapeptide kallidin, are cleaved from kininogen by tissue / plasma kallikrein. Kinins are metabolized rapidly by kininase. Their half-life in plasma is less than 1 min under normal conditions. Kininase activity is decreased in acidic conditions, which may influence the increased kinin concentration during inflammation. Injection of bradykinin into the hind paw produces severe ongoing pain and hyperalgesia [22]. There are two bradykinin receptors (B1 and B2) in primary afferent neurons [23, 24]. Both of them are G-protein coupled. While the B2 receptor is constantly expressed, the B1 receptor does not exist in naïve primary afferents, but increases after tissue inflammation. B2 receptors play an important role in acute nociception, because their antagonists diminish the nocifensive reaction following formalin, acetic acid, acetylcholine and capsaicin injection. Blocking B2, but not B1 receptors, alleviates plantar incision-induced postoperative pain hypersensitivity [25]. Bradykinin induces protein kinase C (PKC) ε [26] and thereby sensitizes primary afferent neurons by activating TRPV1 [26, 27], TRPA1 [28] and sodium channel Nav1.9 [29].

## PROSTAGLANDIN E2

Prostaglandin E2 (PGE2) is one of the major prostanoids derived from arachidonic acid. Two cyclooxygenase enzymes, COX-1 and COX2, mediate biosynthesis of PGE2 [30]. COX-1 is expressed constitutively in various kinds of tissue. COX-2 does not exist in normal tissue but is induced by pro-inflammatory cytokines and growth factors. PGE2 concentration in the skin tissue decreases after the injury that is parallel to the decline of COX-1 expression [31]. By contrast, PGE2 concentration and COX2 expression in the muscle [32] and bone [33] increase after the injury. Four PGE2 receptors (EP1-EP4) have been identified in the primary afferent neurons. All of four receptors are coupled with G-protein but have distinctive intracellular signaling (Table **3**). PGE2 increases capsaicin induced currents in cultured rat DRG neurons [34] and sensitize TRPV1 in cultured mouse DRG *via *activation of PKA and PKCε [35]. Tetrodotoxin (TTX)-resistant sodium current in rat DRG neurons positively regulated by PGE2 [36]. PGE2 induces increase in the magnitude of the peak current and hyperpolarizing shift of current voltage relationship. Gene deletion of two classes of TTX-resistant sodium channels, Nav1.8 [37] or Nav1.9 [29], showed impaired pain hypersensitivity induced by the PGE2 injection.

## PROTEINASE-ACTIVATED RECEPTORS (PAR)

Proteinases, such as thrombin, tryptase and trypsin, generated by tissue injury, activate G-protein coupled proteinase activated receptors (PARs) by cleavage of their N-terminal extracellular domain [38]. Among the four members of the PAR family, PAR1, PAR2 and PAR4 are present in primary afferent neurons. PAR2 is activated by trypsin and tryptase. Injection of trypsin into the paw elicits pain hypersensitivity [39]. Activation of PAR2 is associated with thermal hyperalgesia *via *sensitization of TRPV1 [40, 41] and mechanical allodynia *via *TRPV4 activation [42]. PAR2 also activates P2X3 current *via *activation of PKA and PKCε pathway [43]. 

PAR1 is activated by thrombin. Intra-plantar injection of thrombin increases the nociceptive threshold and diminishes inflammation induced pain hypersensitivity [44]. The anti-nociceptive effect of PAR1 is involved in opioid signaling, since treatment with PAR1 agonists increases proenkephalin mRNA expression, and its analgesic efficacy is reversed by the opioid receptor antagonist, naloxone [45]. Activation of PAR4 also exerts anti-nociceptive effects against tissue inflammation and visceral hypersensitivity [46, 47].

## CYTOKINES

Local treatment with proinflammatory cytokines, including Interleukin (IL)-1β [48], tumor necrosis factor (TNF)-α [49] and IL-6 [50], induces hyperalgesia at the site of injection. Cytokines directly sensitize primary afferent neurons *via *their receptors [50-53]. The majority of nociceptors in the DRG have cytokine receptors, including the IL-1 receptor [51], TNF receptor 1 and 2 [52] and IL-6 receptor gp130 [24]. TNF-α activates TRPV1 and TTX-resistant sodium channels *via *a p38 mitogen-activated protein kinase (p38 MAPK) [54, 55] and PKCε [56] dependent mechanism. We recently showed that p38 MAPK is activated in the primary afferent neurons after a plantar incision in a TNF-α dependent manner. IL-1β increases excitability by sensitizing TTX-resistant slow and persistent sodium channels by activating p38MAPK [51]. In addition to the direct mechanism, IL-1β leads to the synthesis of prostaglandins [57] and nerve growth factor (NGF) [58], which cause prominent pain hypersensitivity [59].

### Nerve Growth Factor (NGF) and Other Tropic Factors

While NGF has been considered to work pro-nociceptive, recent investigations have demonstrated that other tropic factors, including glial cell-derived neurotropic factor (GDNF), brain derived neurotropic factor (BDNF) and insulin-like growth factor (IGF), also have pro-nociceptive effects. They sensitize primary afferent neurons *via *tyrosine kinase-linked receptors.

## NERVE GROWTH FACTOR (NGF)

NGF has prominent effects on the sensitization of primary afferent neurons [60]. The receptor for NGF, trk-A, is expressed in the primary afferent neurons. Injection of NGF into the skin causes immediate hyperalgesia [61, 62]. NGF promotes pain hypersensitivity by activating TRPV1 [63], TRPA1 [64], B1 bradykinin receptors [65], TTX-resistant sodium channels and potassium channels [66]. NGF action can be regulated not only by modulating their function, but also by increasing their expression. Tissue inflammation increases TRPV1 positive neurons by NGF-dependent mechanisms [63, 67]. Tissue injury increases NGF synthesis [68] and NGF inhibition alleviates thermal hyperalgesia after incision [69].

### Brain-derived Neurotropic Factor (BDNF)

BDNF has a prominent role for the sensitization of the sensory neurons in the spinal cord after the tissue inflammation or nerve injury. Compared to spinal effect, role of BDNF on the peripheral sensitization has been less extensively studied. 

The expression of BDNF in the DRG increased after the tissue inflammation [70] or nerve injury [71]. Trk-B, a selective receptor for the BDNF, is expressed in the primary afferent neurons as well. Approximately 10% of primary afferent neurons are positive for trk-B [72] and the expression increased by the tissue inflammation [73]. In cultured neurons, BDNF induces phosphorylation of ERK [73] that subsequently sensitize the primary afferent neurons during the tissue inflammation. Tissue injury induces up-regulation of BDNF and anti-BDNF treatment reduces tissue injury induced pain hypersensitivity [74].

## GLIAL CELL-DERIVED NEUROTROPIC FACTOR (GDNF)

GDNF, neurturin, artemin and presepin are members of the GDNF family. Correspondingly, four receptors have been identified: GDNF preferentially binds to GDNF-family co-receptor α1 (GFRα1), neurturin to GFRα2, artemin to GFRα3 and presepin to GFRα4 [75]. GDNF, neurturin and artemin potentiate the function [76] and expression [77] of TRPV1 after inflammation. Injection of GDNF, neurturin and artemin into the hind paw produces hyperalgesia against heat [76]. The concentration of artemin and GDNF increases in injured tissue after paw incision [78].

## INSULIN-LIKE GROWTH FACTOR (IGF1)

IGF1 is synthesized in dermal fibroblasts and organizes epidermal growth and differentiation [79]. IGF also promotes proliferation of Schwann cells and facilitates myelination of peripheral nerves [80]. IGF1 synthesis is increased after tissue injury to facilitate wound healing. Receptors for IGF1 (IGFR1) have been identified in the primary afferent neurons. IGF1 activates TRPV1 function in cultured primary afferent neurons [81]. We demonstrated that local injection of IGF1 promotes pain hypersensitivity. Tissue injury increases IGF1 concentration and IGF1 inhibitors alleviate the hyperalgesia after tissue injury [82].

## CONCLUSION

In this review, we discussed the mechanisms by which tissue injury drives pain hypersensitivity at the peripheral level. Various mediators, including intracellular algogenic factors, proinflammatory cytokines, kinins and tropic factors, directly activate primary afferent neurons. These factors are also capable of exciting immune cells, increasing their own production and/or that of other classes of mediators. Blocking the action of peripheral mediators at the site of injury could be a promising mechanism-based therapeutic approach to prevent pain hypersensitivity.

It is worthwhile to note that in some conditions, in addition to the peripheral mechanisms, abnormalities in central components, including the spinal cord, brainstem and higher brain structures, are involved in the pathophysiology of pain hypersensitivity after tissue injury.

## Figures and Tables

**Table 1.** Intracellular Algogenic Factors and their Receptors in the Primary Afferent Neurons

	ATP	Glutamate	Proton
ionotropic receptors	P2X (P2X2, P2X3, P2X4, P2X5, P2X6, P2X7)	AMPA, NMDA, Kainate receptor	ASIC3
metabotropic receptors	P2Y (P2Y1, P2Y2)	Group I-III mGluRs	

**Table 2.** Inflammatory Mediators Affect to the Primary Afferent Neuron

	Bradykinin	Proteinase	PGE2	TNF-α	IL-1β

Receptors	B1*, B2	PAR1**, PAR2 PAR4**	EP1-EP4	TNFR1	IL-1R
				TNFR2	

Intracellular signaling	PKCε	PKA	PKA	p38 MAPK	P38 MAPK
		PKCε	PKCε	PKCε	

Biological action	TRPV1↑	(PAR2)	TRPV1↑	TRPV1↑	TTX-r VGSC↑
	TRPA1↑	TRPV1↑	TTX-r VGSC↑	TTX-r VGSC↑	
	nav1.9↑	TRPV4↑			

* Inducible, does not exist in naive

** anti-nociceptive. VGSC: Voltage-gated sodium

**Table 3.** PGE2 Receptors and their Intracellular Signaling

	EP1	EP2	EP3	EP4

G-protein	Gq/11	Gs	Gi	Gs

Intracellular action	PKCε↑	cAMP↑	cAMP↓	cAMP↑
		PKA↑	Ca↑	PKA↑
